# Conceptualising research environments using biological niche concepts

**DOI:** 10.1007/s13194-025-00640-w

**Published:** 2025-03-01

**Authors:** Rose Trappes, Sabina Leonelli

**Affiliations:** 1 Egenis Centre for the Study of the Life Sciences, University of Exeter, Byrne House, St German’s Road, Exeter, EX4 4PJ UK; 2 Centre for the Study of the Sciences and the Humanities, University of Bergen, Bergen, Norway; 3 Department of Science, Technology and Society, Technical University of Munich, München, Germany

**Keywords:** Niche construction, Cognitive niche, Epistemic niche, Scientific change, Conceptual ecology, Nersessian, Nancy, Rouse, Joseph

## Abstract

Several philosophers of science have taken inspiration from biological research on niches to conceptualise scientific practice. We systematise and extend three niche-based theories of scientific practice: conceptual ecology, cognitive niche construction, and scientific niche construction. We argue that research niches are a promising conceptual tool for understanding complex and dynamic research environments, which helps to investigate relevant forms of agency and material and social interdependencies, while also highlighting their historical and dynamic nature. To illustrate this, we develop a six-point framework for conceptualising research niches*.* Within this framework, research niches incorporate multiple and heterogenous material, social and conceptual factors (multi-dimensionality); research outputs arise, persist and differentiate through interactions between researchers and research niches (processes); researchers actively respond to and construct research niches (agency); research niches enable certain interactions and processes and not others (capability); and research niches are defined in relation to particular entities, such as individual researchers, disciplines, or concepts (relationality), and in relation to goals, such as understanding, solving problems, intervention, or the persistence of concepts or instruments (normativity).

## Introduction

Philosophers use various frameworks to understand the circumstances of knowledge creation and circulation. In this paper we examine three approaches that use biological concepts of niche, what we call niche-based theories of scientific practice: *conceptual ecology* (Griffiths & Stotz, 2008; Stotz & Griffiths, 2004), *cognitive niche construction* (MacLeod & Nersessian, 2013; Nersessian, 2008, 2022), and *scientific niche construction* (Rouse, 2015). We compare and build on these three theories, using conceptual resources from biology to develop an overarching framework for conceptualising research niches.

Within this framework, research niches incorporate multiple and heterogenous material, social and conceptual factors (multi-dimensionality). Research outputs arise, persist and differentiate through interactions between researchers and these multiple and heterogenous factors (processes), with researchers actively responding to and making changes to niches (agency), and conditions enabling as well as constraining certain interactions and processes and not others (capability). Finally, research niches are defined in relation to particular entities, including individual researchers, disciplines, communities, concepts, or research programmes (relationality); and they are defined in relation to certain goals, such as understanding, solving problems, intervention, or the persistence of concepts or instruments (normativity).

This six-part framework systematises and extends niche-based theories in philosophy of science, setting up the research niche as a promising concept for understanding scientific practice and highlighting its potential for exploring relevant forms of agency and material and social interdependencies while emphasising their historical and dynamic nature. It is not our aim to argue for niche-based theories as indispensable for investigating scientific knowledge production, and our analysis points to some of the limitations and dangers of biological analogies. What we contribute is a systematic, up-to-date assessment of what contemporary niche concepts, as used in biology and philosophy, can do for the general philosophy of science, and under which conditions.

We start in Sect. 2 by introducing the focus on contexts and conditions of research and summarising biological niche concepts and theories. In Sect. 3 we outline three philosophical uses of niche concepts to theorise research practice. Section 4 highlights commonalities and differences amongst those three niche-based theories of scientific practice and proposes a six-point framework to conceptualise research niches. In Sect. 5 we discuss how such a framework can help to conceptualise and investigate the complex and dynamic conditions of scientific research. We briefly conclude in Sect. 6.

## Exploring scientific research environments

Many philosophers have recognised the importance of place, location, or context for knowledge making practices. In this section we introduce discussions from feminist philosophy; practice-based philosophy of science; and historical, cognitive and social studies of science. We also begin to introduce the niche as a conceptual tool, providing some background on the niche concept in biology.

### Thinking about contexts and conditions of research

Feminist philosophers have long argued for the situatedness of knowers and knowledge, and for the dependency of knowledge making and sharing on social and material contexts (Collins, 1986; Haraway, 1988; Harding, 1991; Longino, 2002; Rolin, 2011). For example, bell hooks argues that marginality can be an intentionally occupied and cultivated space—for instance by academics from poor black communities—and thereby serve as “a central location for the production of a counter hegemonic discourse” (hooks, 1989, 20). Similarly, standpoint theorists argue that social, cultural, economic and political factors shape the knowledge that communities acquire and the knowledge-making practices they engage in (Harding, 1991, 2015; Intemann, 2010; Wylie, 2012). More generally, feminist epistemologists have argued for the embeddedness of knowers in social and material relations and the importance of attending to these relations in considering knowledge-making and its consequences (Code, 2006).

In conversation with and partly growing out of feminist philosophy, practice-based philosophy of science involves “paying close attention to the *specific contexts* in which scientific results are produced, used, and disseminated” (Soler et al., 2014, 18, emphasis in original). Attention to context in turn leads to greater consideration of material, social, and political dimensions of science and their consequences for scientific knowledge (Ankeny et al., 2011; Soler et al., 2014). Many philosophers of science now study how socio-political factors such as funding structures, policy making goals, or the composition of research communities affect scientific research. They also study science as it takes place in labs and fields, hospitals and clinics, in industry and at the policy-making table, online and in the public sphere, and in various locations around the globe.

Philosophers have also taken an interest in the materiality of scientific reasoning. This work takes inspiration from history of science, particularly the history of experimental instruments and materials (Gooding, 1990; Harré, 2003), and philosophy of mind, especially embodied, enacted and embedded cognition (Clark, 2010; Nersessian, 2008, 2022; Sanches de Oliveira et al., 2023). In addition, historians and philosophers of science have considered how interaction with specific objects and locations fosters or inhibits specific ways of thinking and doing (Hacking, 1992, 2012; Pickstone, 2000; Radder, 2003). And of course, scholars in the social studies of science have investigated research activities in their material, social and political contexts, a line of research which is highly consequential for scientific epistemology (Longino, 2002; Pickering, 1995).

There are many concepts available to help capture the nuances and complexities of the surroundings of science. One source of inspiration is biology, a domain replete with conceptualisations of the surroundings of organisms and how they may affect and be affected by the development, evolution, and interactions of individuals and groups, as exemplified by the ideas of climate, *Umwelt*, habitat, environment, ecotype, and niche (Baedke & Buklijas, 2023; Benson, 2020). There is a long history of these concepts being applied in a variety of ways within philosophy, of which we here consider only recent decades.^1^ For example, epistemologists have drawn on concepts of environment, niche, and landscape to capture phenomena such as learning, fake news and social media, as well as for formal epistemology (Alexander et al., 2015; Arfini et al., 2019; Blake-Turner, 2020; Goldberg, 2020; Levy, 2018; Marin, 2022; Ryan, 2018; Weisberg & Muldoon, 2009). In addition, philosophers of mind have cast 4E approaches to cognition (including knowing) as consisting in the interaction of an agent with relevant elements of its environment (Clark, 2006; Shapiro & Spaulding, 2021).

Here we focus on the theoretical resources of the *niche* concept. A number of philosophers of science have drawn on the niche concept to characterise scientific practice. For instance, Thomas Kuhn drew on the concept to make sense of how epistemic values and goals shape scientific theory (Kuhn, 1990), David Hull used niche theory to characterise the impact of scientists’ social interactions (Hull, 1988), and Chris Haufe has recently drawn on niche construction theory to account for how scientific communities select and refine scientific practices (Haufe, 2022). In the next section, we consider three recent niche-based theories of scientific practice: conceptual ecology (Griffiths & Stotz, 2008; Machery et al., 2019; Stotz & Griffiths, 2004), cognitive niche construction (Nersessian, 2022) and scientific niche construction (Rouse, 2015). But to begin with, why have niches proven so profitable for practice-based philosophers of science? How does thinking about the niche help explore the embodied, agential, material, social, discursive and conceptual elements of science and scientific change? We can begin to answer these questions by looking more closely at the niche as it is theorised in biology.

### The niche concept in biology

The niche has been defined, theorised, and investigated in various ways since its inception in early twentieth century ecology (Griesemer, 1992; Pocheville, 2015). There are many applications of the concept to describe and explain species distribution and coexistence, resource use, and so on (Elith & Leathwick, 2009; Kearney, 2006; McInerny & Etienne, 2012b). It has also travelled beyond ecology to fields as diverse as evolutionary biology, cognitive science, business studies, and sustainability science.

The niche concept generally refers to a kind of relation or fit between an organism (or other entity) and its environment. Three features make the concept especially suitable for theories of scientific practice. First, the niche is multidimensional, incorporating many different dimensions, factors, or elements in the environment. Second, the niche concept is employed for describing and explaining many ecological and evolutionary processes, such as persistence, differentiation, adaptation and dispersal. Finally, theorising about niches often highlights the ways in which organisms can respond to or induce changes in their niches.

These three features are evident in fundamental theorising about ecological niches, such as G. Evelyn Hutchinson’s influential definitions of the fundamental and the realised niche (Hutchinson, 1957, 1978). The *fundamental niche* includes all sorts of ecological conditions, both abiotic and biotic, under which a population or species could persist indefinitely. For instance, the fundamental niche of a species of lake fish might include a temperature range and range of prey sizes, as well as many other dimensions. The *realised niche* is in turn the conditions under which the population or species actually is persisting, typically a subset of the fundamental niche delimited by factors such as interspecific competition (Hutchinson, 1957). For instance, in response to interspecific competition, a population may consume less preferrable resources, thereby changing its realised niche to minimise competition.

Other biologists have defined further niches in order to pick out ecological factors that play roles in other biological processes. For instance, Robert Holt distinguishes the *establishment niche*, the conditions under which a population could first become established, from the *persistence niche*, the conditions under which a population could persist once established (Holt, 2009). The *evolutionary niche* of niche construction theory includes all the selection pressures to which a population is exposed, that is, all the ecological factors that differentially affect survival and reproduction of individuals in a population (Odling-Smee et al., 2003, 40; Trappes, 2021). The *developmental niche*, in contrast, picks out those factors that are relevant to a developing organism, which often differ from the conditions required in adulthood (Stotz, 2017). Biologists have also introduced concepts like the *individualised niche*, picking out the ecological conditions relevant to a particular individual’s survival and reproduction, and the *social niche*, a subset of the individualised niche including just intraspecific interactions (Kaiser & Morrow, 2025; Saltz et al., 2016; Takola & Schielzeth, 2022; Trappes et al., 2022).

Sometimes, niches are understood as pre-existing, static “spaces” in ecosystems which species occupy and to which they adapt through evolution. However, more recently biologists have emphasised the dynamic nature of niches in how entities respond to and alter their niches. Particularly in response to a sub-optimal realised niche, an entity can change itself (via plasticity or evolutionary adaptation) or its environment (via niche construction or niche choice). This is one of the major insights behind niche construction theory as well as other theoretical frameworks capturing how organisms adapt to and adjust their environments (Aaby & Ramsey, 2022; Baedke et al., 2021; Edelaar et al., 2017; Lewontin, 2000; Odling-Smee et al., 2003; Trappes et al., 2022).

Despite controversies over its usefulness (Justus, 2019; McInerny & Etienne, 2012a; Wakil & Justus, 2022), the niche concept plays a number of important epistemic roles. These include serving descriptive and explanatory goals, such as describing the ecological requirements of a population or explaining species co-existence or invasion potential. The niche concept also plays other epistemic roles: being broad and relatively abstract, while still grounded in consideration of the material features of biological agency, it can shape research agendas and is involved in integration of disciplinary perspectives and disparate phenomena (Kaiser & Morrow, 2025; Trappes, 2021).

## Three niche-based theories of scientific practice

In this section we introduce three uses of the niche concept to theorise scientific practice: conceptual ecology, cognitive niche construction, and scientific niche construction. These theories by no means amount to a unified or consistent approach. They were independently developed, and they differ in their details and focus: scientific concepts, practices, researchers, or disciplines are seen to construct, occupy, compete for or adapt to epistemic or cognitive niches. Nevertheless, looking at these theories together brings to light a shared appreciation of the wide variety of conditions of scientific practice as well as attention to scientific change and plurality.

### Conceptual ecology

The first niche-based theory of scientific practice we consider is *conceptual ecology*, developed most prominently by Karola Stotz and Paul Griffiths (Griffiths & Stotz, 2008; Stotz & Griffiths, 2004). Conceptual ecology arose as a way to make sense of conceptual change, and especially the diversification of scientific concepts.

Griffiths and Stotz argue that concepts diversify as new phenomena are discovered and new techniques developed to study those phenomena. They view this as a process of adaptation, “in which concepts become adapted to specific epistemic niches.” (Griffiths & Stotz, 2008, 1). Epistemic niches, in turn, consist of the theoretical and practical needs of investigators given their research goals and the phenomena under study, the technology and skills available, and so on (Griffiths & Stotz, 2008, 2). In other words, conceptual ecology theorises how scientific concepts become adapted to suit local research environments, and how new conditions such as technology can lead to conceptual diversification or “speciation” (Stotz & Griffiths, 2004, 6; Stotz, 2009, 236).

Stotz and Griffiths initially elaborated their theory of conceptual ecology in the context of their empirical research on gene concepts around the turn of the millennium. In this work, they administered questionnaires to practising biologists in order to probe their use of different gene concepts. They had a particular focus on whether and how concepts differ between research communities. For instance, they asked whether molecular biologists, evolutionary biologists, and developmental biologists differed in their identification of genes with causes underlying heritable traits, with particular DNA sequences, or with certain functional elements of the genome (Stotz & Griffiths, 2004).

In characterising and explaining the conceptual differences they predicted and observed, Griffiths and Stotz utilised the concept of the *epistemic niche*:

The Representing Genes project was in part an attempt at ‘conceptual ecology’, that is, an attempt to determine some of the pressures that caused the gene concept to diversify into a number of different epistemic niches. Newly discovered phenomena have necessitated new conceptions of the gene, but the new conceptions have not displaced earlier conceptions, which often remain best adapted to the classes of genetic phenomena which they were devised to handle. As a result, multiple conceptions of the gene have come to coexist… (Griffiths & Stotz, 2008, 10)

In addition to the discovery of new phenomena, Griffiths and Stotz argue that research methods and technology as well as research goals affect conceptual change and diversification. The techniques of classical genetics, such as heredity studies, require a functional definition of the gene as the cause of a heritable trait; this is still the gene concept used by medical geneticists, for instance, whose aim might be to identify the gene causing a particular disorder. In contrast, the molecular gene concept arose to suit the goals and sequencing technology available to late twentieth century molecular biologists and is therefore localised to a particular sequence of DNA. Looking ahead, Griffiths and Stotz predict that the development and widespread implementation of software for automated gene annotation may lead to further changes in the gene concepts used by biologists—“a fine example of […] the demands made on concepts by a changing epistemic niche” (Griffiths & Stotz, 2008, 12).

In their work on genes, Stotz and Griffiths focus on processes of adaptation and specialisation of concepts in response to the pressures in their epistemic niches—target phenomena, available technology, research goals, and so on. In more recent research together with Edouard Machery, Griffiths and Stotz apply their conceptual ecology approach to the concept of innateness (Machery et al., 2019). There, they find that some concepts—in particular innateness—persistently fail to become adapted to their epistemic niches, that is, they are not suited for researchers’ goals and available technologies and skills.

Griffiths and Stotz build explicitly on Hull’s evolutionary epistemology (Hull, 1988; Machery et al., 2019, 180). In his study of the history of taxonomy, Hull argued that research groups create “conceptual niches” that allow them to develop and disseminate new conceptual systems, niches which he associated with the researchers’ target phenomena (Hull, 1988, 395). Moreover, Hull suggested that research groups with similar niches (i.e., similar target phenomena) engage in competition, which they can reduce by differentiating their target phenomena.

Several other philosophers of science have suggested similar mechanisms. For instance, Fridolin Gross, Nina Kranke and Robert Meunier have argued that competition between research groups or disciplines studying the same phenomenon can lead to specialisation (Gross et al., 2019). Rather than adapting concepts to suit an existing epistemic niche, then, the authors suggest that researchers may construct new epistemic niches: “If one wishes to employ an ecological metaphor (or economic for that matter), epistemic competition facilitates the construction of separate niches” (Gross et al., 2019, 4). Similarly, Stefan Linquist suggests that disciplines can acquire distinct epistemic niches by developing explanatory practices that serve different but complementary explanatory roles or functions, such as ecology and evolutionary biology (Linquist, 2019). This also aligns with recent work on scientific concepts and their diversification and adaptation to different research domains (Novick, 2023).

Griffiths’ and Stotz’s conceptual ecology can fairly neatly encompass these ideas about epistemic competition and disciplinary specialisation: conceptual and theoretical elements can be adapted to suit new epistemic niches (target phenomena, technologies, skills, etc.), but researchers may also construct new epistemic niches (new research goals, etc.) to minimise competition with other researchers or disciplines and their conceptual-theoretical apparatuses, or to distance themselves from perspectives that may not suit their findings, circumstances or intuitions (as in the case of innateness).

### Cognitive niche construction

The second niche-based theory we consider is *cognitive niche construction* as developed by Nancy Nersessian (MacLeod & Nersessian, 2013; Nersessian, 2008, 2022). Like conceptual ecology, cognitive niche construction examines the way research conditions shape conceptual and theoretical developments in science. But rather than evolutionary epistemology, cognitive niche construction takes environmental perspectives in cognitive science and 4E cognition as its starting point. In addition, this approach considers more explicitly the agency of researchers in constructing cognitive niches.

Nersessian is well known for applying environmental perspectives on cognition to modelling and concept creation in science (Nersessian, 2008, 2022). “Environmental perspectives” in cognitive science—especially theories of embodied, enculturated, distributed, or situated cognition—make human action the focal point for understanding cognition and emphasize that social, cultural, and material environments are integral to cognition (Nersessian, 2008, 8). Nersessian uses these theories to argue that scientific reasoning involves the interactions amongst researchers and elements of their surroundings. As she writes,

Although it is possible that simple model-based reasoning could take place only “in the head,” reasoning of the complexity of that in science makes extensive use of external representations. A wide range of data—historical, protocol, and ethnographic—establishes that many kinds of external representations are used during scientific reasoning: linguistic (descriptions, narratives, written and verbal communications), mathematical equations, visual representations, gestures, physical models, and computational models. (Nersessian, 2008, 92)

Hence, modelling and other conceptual activities in science are embodied and extended into complex social, cultural and material environments.

Nersessian developed this approach further in work with Miles MacLeod on model-based reasoning in systems biology (MacLeod & Nersessian, 2013, 2016). Utilising insights from a large ethnographic study of computational and integrated systems biologists working in two different labs, Nersessian and MacLeod consider how systems biologists work in relatively unstructured but constrained task environments. In these “adaptive problem spaces”, systems biologists have to develop new modelling frameworks to solve the modelling problems they are grappling with.

This process of building a framework to structure the problem space is, they argue, a form of “cognitive niche construction” (MacLeod & Nersessian, 2013, 35). They associate this with Andy Clark’s concept of cognitive niche construction, according to which embodied agents build structures such as language to enhance or “scaffold” their ability to think and reason (Clark, 2006). For systems biologists, cognitive niche construction requires manipulating and adapting to a wide array of constraints, including: the biological problem and its complexity; what existing knowledge, infrastructure, and data are available; whether there is access to sufficient funding; computational constraints; the different time scales of wet lab and computational activities; the ability to communicate and collaborate across disciplines; and cognitive constraints in dealing with system complexity (Nersessian, 2022, 179–80). Through modifying and adapting to these constraints, systems biologists are able to develop successful models of the biological systems they are studying.

Cognitive niche construction occurs at multiple levels. It includes individual researchers developing models for specific biological systems, but it also covers lab directors creating broader frameworks for the integration of engineering approaches into biological modelling in their labs (MacLeod & Nersessian, 2013, 47). More recently, Nersessian has argued that research labs themselves, including both researchers and their methods and models, can be seen as distributed cognitive-cultural systems that engage in the cognitive activity of model-based reasoning (Nersessian, 2022, 3). Moreover, different systems biologists and different labs constructed their cognitive niches in different ways, leading to methodological and conceptual innovation.

According to MacLeod and Nersessian, the construction of new cognitive niches is key for progress in systems biology. As they write, “Ultimately what is driving this innovation is a determination to construct new *cognitive niches* in the form of functional model building frameworks that integrate systems biology within the biological sciences” (MacLeod & Nersessian, 2013, 35). In the process of this cognitive niche construction, both the “problems” (the systems and phenomena to be modelled) and the modelling methods are adapted to one another in their new interdisciplinary research environment (MacLeod & Nersessian, 2016, 411). This enables researchers to model the systems they are interested in, and therefore ultimately to understand and control these systems. As it turned out, systems biology emerged as an independent and successful discipline through this niche constructive effort (Nersessian, 2022).

Cognitive niche construction and conceptual ecology are not identical, but we can draw out some interesting similarities. Both consider the mutual adjustment between elements of the research process: for conceptual ecology this is concepts and target phenomena, technologies, and so on; for cognitive niche construction, it is problems and modelling methods, including material, social, linguistic, and conceptual elements. Both also tie this process of adjustment to scientific change, especially the emergence of specialisations but also the development of new techniques, methods, and models.

### Scientific niche construction

The third niche-based theory of scientific practice we consider is Joseph Rouse’s work on scientific research as material and behavioural niche construction (Rouse, 2015, 2016). Like cognitive niche construction, Rouse’s theory of scientific niche construction centralises researchers and their agency in shaping their material, sociocultural, and conceptual environments to best support cognitive activities such as science. However, Rouse develops this theory by drawing on evolutionary niche construction theory.

Rouse aims to account for scientific understanding as a natural phenomenon, coextensive with other human activities that can be scientifically explained (Rouse, 2015, 6). To develop this account, he uses niche construction theory. According to niche construction theory, organisms modify their own niches and the niches of other organisms, thereby affecting evolutionary processes by altering selection pressures as well as by potentially introducing an additional, non-genetic form of inheritance (Odling-Smee et al., 2003; Laland et al., 2016; see also Sect. 2.2). Many theorists have applied niche construction theory to human evolution, including to the evolution of complex cultural, social, and linguistic practices (Heras-Escribano, 2020; Laland & Sterelny, 2006). Rouse extends this by arguing that scientific practice is also an instance of human niche construction: “an ongoing reconfiguration of our socially, discursively, and materially articulated environmental niche” (Rouse, 2015, 217).

Rouse emphasises that scientific practice involves making changes to our material, social, and discursive environments such that we can observe and study new phenomena. Rouse especially considers the example of scientists working in laboratories or experimental fields, who construct *experimental systems*. Experimental systems include physical equipment and material transformations of the world, but also practical skills and their execution as well as social practices or ways of life. All of these elements working together allow certain phenomena to become manifest. As Rouse writes, “Experimental systems are novel rearrangements [of the world] that allow some aspects of the world that are not ordinarily manifest and intelligible to *show* themselves clearly and evidently” (Rouse, 2015, 295).

For instance, Rouse considers the creation of the *Drosophila* experimental system: the adoption of the fruit fly *Drosophila melanogaster* as a model organism for genetics in the early twentieth century (Rouse, 2016, 299–304). As historians of biology have shown, creating this system required sourcing and manipulating biological material, an array of skills and techniques, and certain social arrangements to pass on not only research findings but also those materials, skills and techniques. Moreover, resonating with the work of Stotz and Griffiths, Rouse argues that this experimental system shaped the gene concepts scientists utilised and more generally what they recognised as traits and genetic loci.

According to Rouse, scientists creating experimental systems (such as the *Drosophila* system) are engaging in niche construction. Scientific niche construction involves the material, social and discursive reconfiguration of the world. By altering the world in this way, scientists build on and extend more everyday examples of human niche construction such as building houses and roads or using language. Like these everyday examples, scientific niche construction alters our “socially, discursively, and materially articulated environmental niche” (Rouse, 2016, 217) and thereby, Rouse argues, our possibilities for understanding and interacting with the world.

Viewing scientific practice as niche construction has a number of consequences. First, it highlights the material, social, and embodied dimensions of science. Second, science as niche construction is part of a broader suite of activities that shape our material, social and conceptual niche (Rouse, 2015, 211). For instance, Rouse highlights how science is embedded in institutions and entangled with industrial and commercial actors and processes. Third, viewing scientific practice as niche construction requires shifting from a retrospective to a prospective view, recognising that science is motivated by future research opportunities rather than only past achievements (Rouse, 2015, 211). Acknowledging all of these dimensions, Rouse argues, will produce both a more coherent naturalism and a richer and more detailed grasp of scientific understanding and scientific practice (Rouse, 2015, 217).

These insights about scientific niche construction have recently been elaborated and combined with perspectives from embodied and enactive cognition, resulting in a theory of scientific practice as ecological-enactive co-construction (Sanches de Oliveira et al., 2023). Like Rouse’s account, this perspective emphasises the continuity between scientific practice and other forms of biological niche construction. In addition, it stresses the embodied nature of cognitive processing and the fact that humans are not the only agents involved in scientific research. As Guilherme Sanches de Oliveira, Thomas van Es, and Inês Hipólito write, “scientific knowledge arises through relations of sensitive adaptivity that hold between not only individuals and groups of people using instruments, but also cells, insects, forests, and whatever else scientists in different disciplines study” (Sanches de Oliveira et al., 2023, 21). And it’s not just living objects of study: machines and instruments too exert agency in scientific practice (Pickering, 1995; Rouse, 2015).

**Table:** 

Box 1. Three Niche-Based Theories of Scientific Practice
(1) Conceptual Ecology (Stotz & Griffiths) Researchers adapt concepts to epistemic niches, made up of theoretical and practical needs (e.g., target phenomena, available technology and skills). Changes in epistemic niches induce conceptual diversification. Key example: diversification of gene concepts in the post-genomic age (2) Cognitive Niche Construction (Nersessian) Scientists create cognitive niches, including material, social, linguistic and conceptual elements. Cognitive niches make problems solvable by actively distributing cognitive processes and transforming methods, data, representations, etc. Key example: systems biologists building models of complex biological systems (3) Scientific Niche Construction (Rouse) Scientific practice alters our social, discursive and material niche. This reconfigures conceptual spaces so that the world shows itself in new ways and we change our interactions with the world. Key example: scientists creating experimental systems

## A framework for research niches

Conceptual ecology, cognitive niche construction, and scientific niche construction share many features and motivations, but they also differ in a number of ways (see Box 1). In this section we compare and extend these theories to develop a six-part framework which highlights the overarching fruitfulness of the niche concept for philosophy of science, while also acknowledging its limitations.

First, all three theories use the niche concept to capture *multiple and heterogenous material, social and conceptual factors* in science. The epistemic niche in conceptual ecology includes the technology and skillsets available in a research group or collaboration, as well as the goals of researchers and the biological phenomena under study. The cognitive niche of cognitive niche construction includes a wide array of linguistic, mathematical, visual, physical, and computational elements, as well as the gestures, discussions, and interactions amongst researchers and between researchers and their models. And the biological niche of scientific niche construction includes equipment, skills, social practices, discourse, concepts, and research objects. Although they differ in emphasis, these three niches identify material, social and conceptual elements as equally important for scientific practice. Niche-based theories therefore partake in the core idea of practice-based philosophy of science: that science is more than abstract theories evaluated in a logical space, but is rather embodied, material, social, practical, skilled, and entangled with various socioeconomic and cultural institutions and processes (Ankeny et al., 2011; Soler et al., 2014). Niches provide a conceptual tool to recognise these diverse elements of scientific practice. This aligns with the quest to break down barriers between conceptual, material and social components of research in theorising about science (Longino, 2002).

Second, the three theories share attention to *processes* such as differentiation and construction, thereby reflecting the dynamic nature of research practice as discussed in Sect. 2.1. Conceptual ecology considers how scientific concepts arise, change and differentiate in response to changes in epistemic niches. Cognitive niche construction captures how scientific problems are developed, adjusted, and solved through reasoning processes that construct and adapt a cognitive niche. And scientific niche construction looks at how new possibilities for scientific understanding arise through activities that change our biological niche. Niche concepts and theories provide the means to characterise how scientific concepts, models or understanding arise, persist and differentiate through interaction between researchers and their material, social and discursive surroundings.

Third, these three niche-based theories of scientific practice all recognise the significance of the *agency* of researchers responding to and altering their environments. Conceptual ecology highlights how researchers modify their concepts to suit new epistemic niches: when biologists use terms in different ways, they may be actively shaping their concepts to suit their changing goals, skills, and technology. Cognitive niche construction emphasises how researchers actively construct their cognitive niches in a concrete sense, modifying their physical surroundings to make model-based reasoning possible and effective. Similarly, scientific niche construction focuses on how researchers make changes to their material, social and discursive environments to enable scientific understanding, for instance through creating novel experimental systems or computational devices. The design, iteration and regular revision of institutional, physical and conceptual conditions emerge as a core responsibility for researchers’ plans and practices, and the motor for researchers’ creativity in the long term.

Fourth, and relatedly, all three theories consider how niches *enable certain capabilities and not others*. This is particularly apparent in Nersessian’s cognitive niche construction and Rouse’s scientific niche construction. Both emphasise the way that the niches that researchers create facilitate certain cognitive capacities, enabling researchers to solve problems in particular ways or opening up particular ways of understanding the world – and potentially disregarding alternatives, including the skills required to realise those. Conceptual ecology also considers how the fit between a scientific concept and its niche enables researchers to use certain equipment and study certain phenomena, rather than others. Niches thereby both facilitate and constrain the agency and processes in question. In this sense, niche-based theories are compatible with another metaphor used by philosophers to think about scientific and technological change: that of scaffolds. Scaffolding metaphors have been used to elucidate the changing conditions under which specific ways of doing develop, and the processes through which given elements and assumptions become so entrenched as to become invisible, thereby constituting the background and infrastructure that is taken for granted when approaching a given subject (Caporael et al., 2013; Neto et al., 2023).

In addition to these four common features, there are two additional features of niches which are conceptualised differently in the three niche-based theories of scientific practices we consider. These are brought to light especially effectively when considering biological theorising about niches that we presented in Sect. 2.2.

The fifth important feature is that the niche is frequently conceived as a *relational property*, or at least a relationally defined entity. Unlike an external place or habitat, the ecological niche belongs to an entity as those conditions that it requires or that permit it to flourish. What constitutes the niche thereby changes as the entity in question changes. Niche concepts in other biological disciplines are similarly relational, picking out factors that are relevant to a particular entity, such as a population’s selection pressures or an individual’s developmental needs. As a consequence, when discussing niches it is important to identify the entity to which a niche belongs.

The niche-based theories of scientific practice we discussed above defined epistemic or cognitive niches with respect to different entities. In conceptual ecology, it is concepts which occupy or are adapted to epistemic niches. In contrast, both cognitive niche construction and scientific niche construction focus on human researchers as the niche-bearing entities, but with an important variation in emphasis: while cognitive niche construction tends to take individual researchers (or perhaps research groups) as the entities bearing and constructing niches, scientific niche construction tends to focus on niches constructed by research groups or whole research communities. Clarity around whether concepts, individual researchers, research groups, or research communities are in focus seems central for understanding research environments, given that each entity will depend on and interact with different conditions. In addition, other entities could be thought of as bearing epistemic niches—that is, as relating to (depending on, being adapted to, interacting with, and shaping) a specific set of conditions in a way that enables and influences the way research takes place. Examples include networks or consortia (Andersen, 2016; Leonelli, 2019; Zollman, 2013); data, model or software communities (Parker, 2006; Symons & Horner, 2014); and repertoires or research programmes (Ankeny & Leonelli, 2016; Meunier, 2019). To take one example that has been elaborated in the history of science, Hans-Jörg Rheinberger places epistemic things at the centre of experimental systems, such that experimental systems serve as the immediate environment within which specific entities can contribute to scientific research (Rheinberger, 1997). Within a niche-based framework, experimental systems could be understood as the niches for epistemic things.

Sixth, niche concepts are often *normative* or *teleological*. On many accounts in ecology, niches are not just any conditions that an organism happens to be exposed to, but rather those conditions that support population persistence, individual survival and reproduction, the carrying out of specific ways of life or ecological functions, and so on. Niches, in other words, are supposed to be good or at least tolerable for the niche-bearing entity. Similarly, evolutionary biologists frequently assume that organisms are, on average, adapted to their niches and that they engage in activities which alter their niche to make it more suitable for their survival and reproduction (Odling-Smee et al., 2003, 47; Trappes et al., 2022).

The normativity of niches can help to further distinguish the three niche-based theories of scientific practice discussed above. Conceptual ecology focuses on a fit or match between scientific concepts and the conditions that make up epistemic niches; particularly successful concepts which persist and spread throughout a scientific community are well-adapted to their epistemic niches. For cognitive niche construction, it is the researcher’s ability to solve problems that defines the cognitive niche, and for scientific niche construction niches are constructed to enable understanding of the world. Notably, the goal of truth does not appear in these theories; this aligns with a broader consensus in practice-based philosophy of science that scientists pursue a wide variety of goals but that accuracy does not typically serve as an end in itself (Longino, 2002; de Regt, 2017; Potochnik, 2017).

More generally, we could distinguish a number of different goals with respect to which different niches can be identified, such as scientific understanding; answering research questions; solving problems; theoretical success; identifying and testing interventions; or the development, persistence and spread of a concept, instrument, model, or dataset. Recognising different niches according to different goals of scientific inquiry can advance the theorisation of pluralism. In particular, it connects a diversity of goals with the conditions that facilitate pursuing those goals, without assuming that all researchers pursue one overarching goal such as scientific understanding. It also helps to make sense of situations in which multiple goals are being pursued at once, such as gaining causal understanding and developing accurate predictions to inform policy—a dual objective frequently pursued in research related to public health, as discussed at length by Cartwright and Hardie (2012) and exemplified by the latest case of COVID-19 science (Leonelli, 2021). In these situations, the same set of conditions may be suitable for one goal but not for another.

With relationality and normativity, we now have six core features of niche concepts as they are applied to scientific practice (Box 2). The six-part framework highlights both commonalities and differences amongst the three niche-based theories of scientific practice we discussed above. It also points towards potentials and limitations of niche-based theories of scientific practice more generally.

**Table:** 

Box 2. Six Core Features of Research Niches
(1) *Multi-dimensionality*. Science involves multiple and heterogenous material, social and conceptual factors. (2) *Processes*. Concepts (or models, or scientific understanding) arise, persist, and differentiate via interaction between researchers and their material, social and discursive surroundings. (3) *Agency*. Researchers respond to and make changes in the material, social, and discursive surroundings. (4) *Capability*. Conditions enable certain capabilities (and not others): they make it possible for specific interactions to take place and thereby constrain the agency and processes in question. (5) *Relationality*. Niches are defined with respect to particular entities, including concepts, individual researchers, research groups, disciplines, data communities, or research programmes. (6) *Normativity*. Niches pick out conditions in relation to certain goals, such as understanding, solving problems, intervention, or the persistence of concepts or instruments.

In the next section we consider how this six-part framework can be used to understand and study research environments. Before this, however, we address an important question: just what does it mean to use niche concepts to understand scientific practice?

Some of the philosophers we discussed develop their theories as part of an explicitly naturalist approach. That is, they view scientific practices as continuous with regular cognitive or biological processes and argue that scientific theories and methods should be used to understand science. In particular, Rouse states that his goal is to “advance a naturalistic self-understanding” (Rouse, 2015, 3). Nersessian also sees her cognitive-historical approach as a form of naturalism, holding that philosophical theories should be informed by the best available understanding from biological, cognitive and social sciences as well as historical research, and that empirical methods from these disciplines can be used to develop and test philosophical hypotheses (Nersessian, 2008, 4; 2022, 5).

In contrast, Stotz and Griffiths are more circumspect about naturalism. Although they do use empirical methods, they frequently use scare quotes to refer to “conceptual ecology” and related terms, such as “conceptual phylogeny,” “ecological pressures,” “conceptual speciation,” and “epistemic niche” (Griffiths & Stotz, 2008, 10; Stotz, 2009, 236; Machery et al., 2019, 180). This suggests that they take conceptual ecology to be a more metaphorical use of biological theories and concepts to understand scientific change, or at least that they prefer to remain agnostic with respect to naturalism. Other philosophers of science who have used niche concepts along the lines of conceptual ecology similarly take a metaphorical approach (see Sect. 3.1). On this view, niche concepts are helpful conceptual tools for making sense of the relationship between research and its surroundings.

There are clear risks to bringing ideas from biological research to bear on human social and cultural phenomena, as most clearly instantiated by eugenics and biological essentialism. These are not tendencies we wish to encourage. Nevertheless, philosophers of science find inspiration in biological understandings of coexistence and cooperation under a wide variety of environmental conditions, and we think these understandings may further conceptualisations of how humans operate as inquirers and investigators. Moreover, as decades of historical and sociological research teach us, research environments encompass complex relationships between human and non-human organisms, as well as other non-human agents such as machines. Considering ideas developed in relation to complex biological systems may facilitate thinking of research as including a variety of actors and forms of agency, whose relation to their environment is multifaceted and mutually defining.

This need not be an uncritical rendering of biological analysis into social analysis. On the one hand, the niche concept can be used as a metaphor to inspire a philosophical framing of research conditions. On the other, even a thoroughly naturalist stance does not require the uncritical adoption of all claims and methods from the natural sciences. We cannot here resolve the question of naturalism and the appropriate role of biological concepts in philosophy. However, we hope to make space for a biologically inspired theorisation that is capable of critically evaluating the foundations and implications of biological research.

## Research niches and the complex, dynamic conditions of scientific research

In this section we argue that the niche concept can facilitate the study of scientific practice. It helps draw attention to the complex and dynamic conditions of scientific research, and can provide resources for describing and explaining these conditions and how they affect and are affected by research. In addition, it has the potential to provide an overarching concept to integrate insights about diverse research settings. These epistemic roles align with those of the niche concept more generally.

Philosophy of science has traditionally focused on relatively insular, well-developed, and specialised research. Such research tends to occur in a limited number of fairly homogenous institutions, which have to conform to some basic specifications to count as laboratories or research platforms. These research environments are relatively contained, with specialised, professional individuals assigned to work within them. Moreover, they often have a relatively clear division of labour, anchored and channelled by use of specific technologies, methods and materials that become standardised as part of a well-characterised system of practice (Chang, 2012) and entrenched as “normalised” or “best” practice. This sort of research environment may be easier to study, but it is not representative of the overall diversity of research settings.

First, research settings are increasingly complex, exceeding boundaries and drawing on heterogenous spaces and networks. Through methods such as multi-sited ethnography, cultural geographers and anthropologists have long pointed to the multiplicity and frequent shifts in locations and temporalities involved in scientific work (e.g., Livingstone, 2003; Fischer, 2003; Sunder Rajan, 2021). Many researchers work in and out of laboratories and in a large variety of privately and publicly funded spaces. Research also tends to be geographically and temporally distributed across an often uneven and widely diverse set of locations and schedules. Historical scholarship on multi-species biological investigations and agricultural science is another reminder of how research often spans settings, cultures and systems, giving rise to methods and communities tailored to characteristics of specific targets and goals and embracing various ways of identifying, enacting, institutionalising and assessing human and non-human agency (Kohler, 2002; de Bont, 2015; Curry, 2022; Krige, 2022).

Enhanced by information and communication technologies and international mobility requirements, scientists are often involved in many intersecting groups, networks, and communities, rather than sticking to one organisational unit. These heterogenous groupings bring together multiple interests, concerns, and domains with respect to which scientific research is conducted and evaluated. Under these conditions, scientists juggle the need to extend their networks across institutional, national and disciplinary borders with the necessity of building long-standing, trustworthy collaborations to pursue their overarching goals and support existing connections.^2^

Second, research environments are increasingly dynamic. Research today is subject to frequent changes brought about by shifts in the funding landscape and available resources, and the need to rapidly learn from, respond to and integrate changes in the phenomena at hand, such as climatic or biological conditions (Ankeny & Leonelli, 2016). The modular, innovation-focused and project-oriented science now favoured by most funding bodies around the world involves frequent reshufflings of the kinds of expertise viewed as relevant to research objectives, as researchers are required to create proposals that sound novel and yet help them to pursue their long-term goals (Andersen, 2016). This dynamicity is even present when there are long-term, overarching objectives like the United Nations’ Sustainable Development Goals (SDGs); pursuing these with precarious funding sources often result in bursts of activity under short-term contracts, leading to frequent revisions of research programmes and interactions (Berrone et al., 2023).

The niche concept can help to draw attention to the complexity and dynamicity of research settings, and provides resources for describing and explaining important phenomena. To illustrate, consider the intersections of museums, archives, amateur collectors, biologists and conservation ecologists discussed by Susan Leigh Star and James Griesemer when proposing their foundational concept of “boundary objects” (Star & Griesemer, 1989). In Star and Griesemer’s retelling, a key location is the Museum of Vertebrate Zoology at the University of Berkeley, whose first director Joseph Grinnell was interested in managing biological specimens to facilitate their use for evolutionary biology and ecology. This in turn required processing those specimens in ways that would enable a large variety of relevant experts to access and deploy them for their own purposes.

Star and Griesemer introduce the notion of boundary object to account for the ways that common interest in a specific entity can bring into contact a variety of different spaces, temporalities and forms of expertise (what they call “social worlds”). We could however also interpret this situation as one where a common niche or research environment is created around the deployment of specimens as boundary objects. This niche brought together a wide variety of heterogenous factors (*multi-dimensionality*). Making specimens into boundary objects required carefully articulated interactions amongst researchers and their different institutional, social, and material settings (*processes*). The biologists, collectors, and managers engaged in these processes of creating boundary objects thereby actively shaped their research conditions (*agency*). Although the emergence of a niche around biological specimens does not guarantee that specimens would repeatedly be used in the same way, it does make scientific work on those objects possible: it defines the specific commitments, constraints and opportunities attached to using those objects, without however binding all participants to a uniform set of goals or long-term strategy (*capability*). The work that happens in these settings, and the conditions considered relevant to that work, is tied to and defined by a boundary object (*relationality*). Finally, such work typically includes specific normative commitments, such as shared values or norms around what constitutes a relevant effort or an appropriate goal, though it need not include consensus over exactly which goals to pursue (*normativity*).

As this example briefly indicates, our framework can serve to characterise complex and dynamic research settings. Another role the niche concept can play is in the consideration of what does *not* come together as a system of practice. In other words, how do we make sense of research initiatives failing to take off, become established, or continue? Within a niche-based framework, these kinds of failure can be conceptualised as a lack of fit or match between a project and existing conditions. This is similar to what Chang (2012) discusses as lack of coherence within systems of practice, or what others characterise as misalignments or frictions among different elements of the scientific landscape. The fundamental niche is a useful concept here since it provides a framework to further analyse and identify what conditions are, at least in principle and in ideal cases, suitable or good for a given entity. The fundamental niche can then be compared against the conditions the entity actually experiences: when the conditions fall outside the fundamental niche, the entity will not be able to persist and flourish (see Sect. 2.2). In some cases, the lack of fit may be resolved by adjusting the environment or the entity, for instance through niche construction. But if this cannot be achieved, the entity will fail to become established or persist.

What may be causing a lack of fit between an entity (research group, individual, method, component) and the conditions it is faced with? It could be scientific factors, such as concepts which do not help achieve given purposes (e.g., innateness), methods which are poorly suited to existing expectations (e.g., irreproducible experiments in domains that place a premium on reproducibility), or specific characteristics of the datasets at hand (e.g., where the data are viewed as unreliable or unable to corroborate a given hypothesis). It could also be social, institutional, economic or organisational factors, such as the lack of a suitable venue for potential contributors to gather or the existence of a funding scheme that does not support a given set of activities. Often it will be a mixture of different factors. Hence, a niche-based framework can support a nuanced analysis of scientific change, where what ends up succeeding or failing is assessed with respect to a variety of factors ranging from conceptual to institutional, with varying degrees of significance assigned to such factors depending on the specifics of the situation at hand.

Being such a broad concept, the niche concept may also help to compare and develop general insights about a wide variety of research environments. Consider the cases addressed by the three niche-based theories we examined. Nersessian looks at how systems biologists work between computation, cell biology and biochemistry, with data obtained from literature, collaborators, or their own wet-lab experiments. Rouse mentions a wide range of practices, including theoretical modelling practices, field trials, clinical practices and other modes of empirical and materially transformative engagement with the world. Stotz and Griffiths (and others taking the conceptual ecology approach) also have a broad range of research settings in mind, including the heavily lab-based genetics, molecular biology and developmental biology, but also fields like population genetics with its proximity to both natural history and mathematics and its use of a wide array of technologies often tailored to the specific systems under study.

Using the niche concept for these sorts of examples brings to light key commonalities, such as the interplay between material, social and theoretical factors, the role of human agents and technologies within settings designed to serve as platforms for scientific inquiry, and the dynamic ways in which these settings provide enabling conditions not just for research but for the development of skills and abilities which will fuel research practice in the future. And, given that niches are defined relationally, these sorts of features can be analysed with respect to individual researchers, scientific concepts, research communities, and a range of other entities. The result is a plurality of research niches, each anchored in a specific perspective on scientific practice.

We have sketched several epistemic roles that the niche concept can play, but the niche is of course not the only concept to address the conditions under which research takes place. Social sciences are an especially important location for fundamental contributions to understanding research conditions. Moreover, the niche concept’s proximity to biology may suggest for some that research niches are a given, downplaying ethical and political considerations and avoiding recognition of researchers’ agency and, consequently, their responsibility for shaping and acting under particular conditions. As we have made clear, niches can incorporate social factors and recognise agency. Nevertheless, it may be that alternative concepts, such as those from the social sciences, have resources that enable them to better serve epistemic roles related to for instance assigning responsibility and accountability.

## Conclusion

This paper reviewed philosophical applications of the niche concept to science, considering how the niche concept can support a conceptualisation of research environments as multi-dimensional, processual, agential, enabling, relational, and normative. We argued that this niche-inspired framework can help understand and investigate scientific practice characterised by complex and dynamic threads of dependence on distributed collaborations, materials, tools, software, non-human organisms, funding, concepts, theories, and many other elements of the conditions of research.

The niche concept is one amongst many tools with potential to support studies which reveal the epistemic importance and the complexity and dynamicity of the conditions of scientific practice. Equipping ourselves with these sorts of concepts is crucial to improving our understanding of conditions under which research is conducted and validated. This is in turn an important step towards improving current ways of evaluating the reliability and robustness of scientific claims, by facilitating the appropriate contextualisation of the circumstances of knowledge creation and use across a variety of settings and purposes. This is particularly relevant in relation to the life sciences broadly construed, which take place under a wide array of ever-changing material, social and institutional conditions as the niches of both research subjects (e.g., experimental organisms) and scientists themselves influence and co-construct each other.

## Data Availability

N/A.

## Footnotes

1 There is no overarching review of the use of biological metaphors in philosophy. However, scholars have produced rich analyses of the use of metaphors within natural philosophy and their broader cultural influence (see, e.g., Tauber, 1994; Keller, 1995; Reynolds, 2018).

2 For a detailed discussion and examples of contemporary scientific practices of this type, see the paper by Leonelli and Trappes in this topical collection.
